# The effect of glutamine therapy on outcomes in critically ill patients: a meta-analysis of randomized controlled trials

**DOI:** 10.1186/cc13185

**Published:** 2014-01-09

**Authors:** Qi-Hong Chen, Yi Yang, Hong-Li He, Jian-Feng Xie, Shi-Xia Cai, Ai-Ran Liu, Hua-Ling Wang, Hai-Bo Qiu

**Affiliations:** 1 Department of Critical Care Medicine, Zhong-Da Hospital, School of Medicine, Southeast University, 87 Dingjiaqiao Road, Nanjing 210009, P.R. China; 2 Department of Critical Care Medicine, Su-Bei Hospital of Jiangsu Provience & Clinical Medical School, Yangzhou University, Yangzhou, Jiangsu, P.R. China

## Abstract

**Introduction:**

Glutamine supplementation is supposed to reduce mortality and nosocomial infections in critically ill patients. However, the recently published reducing deaths due to oxidative stress (REDOX) trials did not provide evidence supporting this. This study investigated the impact of glutamine-supplemented nutrition on the outcomes of critically ill patients using a meta-analysis.

**Methods:**

We searched for and gathered data from the Cochrane Central Register of Controlled Trials, MEDLINE, Elsevier, Web of Science and ClinicalTrials.gov databases reporting the effects of glutamine supplementation on outcomes in critically ill patients. We produced subgroup analyses of the trials according to specific patient populations, modes of nutrition and glutamine dosages.

**Results:**

Among 823 related articles, eighteen Randomized Controlled Trials (RCTs) met all inclusion criteria. Mortality events among 3,383 patients were reported in 17 RCTs. Mortality showed no significant difference between glutamine group and control group. In the high dosage subgroup (above 0.5 g/kg/d), the mortality rate in the glutamine group was significantly higher than that of the control group (relative risk (RR) 1.18; 95% confidence interval (CI), 1.02 to 1.38; *P* = 0.03). In 15 trials, which included a total of 2,862 patients, glutamine supplementation reportedly affected the incidence of nosocomial infections in the critically ill patients observed. The incidence of nosocomial infections in the glutamine group was significantly lower than that of the control group (RR 0.85; 95% CI, 0.74 to 0.97; *P* = 0.02). In the surgical ICU subgroup, glutamine supplementation statistically reduced the rate of nosocomial infections (RR 0.70; 95% CI, 0.52 to 0.94; *P* = 0.04). In the parental nutrition subgroup, glutamine supplementation statistically reduced the rate of nosocomial infections (RR 0.83; 95% CI, 0.70 to 0.98; *P* = 0.03). The length of hospital stay was reported in 14 trials, in which a total of 2,777 patients were enrolled; however, the patient length of stay was not affected by glutamine supplementation.

**Conclusions:**

Glutamine supplementation conferred no overall mortality and length of hospital stay benefit in critically ill patients. However, this therapy reduced nosocomial infections among critically ill patients, which differed according to patient populations, modes of nutrition and glutamine dosages.

## Introduction

Glutamine is the most abundant plasma and intracellular amino acid. It is known as an essential nutrient for the gastrointestinal tract during critical illness. The efflux of glutamine from the skeletal muscles serves as a carrier of nitrogen to the small intestine [1]. Increased glutamine use occurs during critical illness, which causes a significant glutamine deficiency and oftentimes results in an impaired immune response to infections [2]. Lower plasma and skeletal muscle glutamine levels have been associated with immune dysfunction [3] and a higher mortality rate in critically ill patients [4].

In animal studies [5], glutamine decreased gut mucosal atrophy when supplemented in the parenteral nutrition that was administered to the animals. In addition, glutamine also reduced bacterial translocation in additional animal models [6]. Some animal studies [7,8] also demonstrated that glutamine supplementation improved survival in experimental models of sepsis.

In a human study [9], supplementation of enteral and parental nutrition with glutamine was observed to improve immunologic function and preserve intestinal morphology and function. In addition, glutamine supplementation may also reduce bacterial translocation [10].

Recent clinical studies [11-13] have suggested that parenteral administration of glutamine to ICU patients reduces mortality and the incidence of new infections. However, these studies were conducted in small trials, many of which were of poor quality. Recently, two large trials [14,15] reported the administration of glutamine supplementation during critical illness, but did not provide similar evidence for a benefit from glutamine supplementation. Heyland *et al*., in the Reducing Deaths due to Oxidative Stress (REDOX) study [14], observed significantly increased in-hospital and six-month mortality rates with the use of glutamine, without reducing the nosocomial infection rate in ICU patients. The aim of this meta-analysis was to examine whether glutamine supplementation in ICU patients reduces mortality, the occurrence of nosocomial infections and the length of hospital stay.

## Material and methods

### Inclusion criteria

We included trials with the following features:

1. Type of studies: randomized controlled clinical trials

2. Population: adult ICU patients

3. Intervention: intravenous or enteral glutamine supplementation

4. Placebo alone or no intervention

5. The following outcomes were included: a) primary outcomes: in-hospital mortality, or if not reported, ICU/28-day/mortality; b) secondary outcomes: six-month mortality, nosocomial infection and length of hospital stay.

### Search strategy for the identification of studies

We conducted a search of the following databases: Medline (1948 to April 2013), Elsevier, Cochrane (Central) database, Web of Science and ClinicalTrials.gov. As search terms for each database, the following keywords were used: ‘glutamine’ or ‘glutamine dipeptides’ or ‘L–glutamine’ or ‘glutamine supplementation’ and ‘critical care’ or ‘critical patients’ or ‘critical ill’ or ‘critically ill patients’ or ‘critical illness’ or ‘serious illness’ or ‘seriously ill’ or ‘intensive care units’ or ‘intensive care’ or ‘surgical intensive care unit or ‘SICU’ or critical care medicine.’ An additional DOCX file shows this in more detail [see Additional file 1].

### Study selection

Two reviewers independently screened titles and abstracts to determine whether a particular study met the inclusion criteria. The full texts of the articles were then reviewed independently according to the inclusion and exclusion criteria. Any discrepancies were resolved by reaching a consensus on the inclusion or exclusion of a particular study following a discussion with a third reviewer.

### Data extraction and management

Two reviewers independently extracted data using a standardized data extraction protocol. Any disagreements between the two reviewers were resolved by a discussion, whereby a consensus was then reached.

Some parameters, such as the mean glutamine dosage, were estimated from other available parameters. Some mean and standard deviations of the patients’ length of hospital stay data were estimated according to the method described by Hozo [16].

### Methodological quality assessment

The Jadad score was constructed by adding the elements of the use of the analysis and the blinded endpoint assessments. For each item from the resulting list, we assigned two points if the criterion was fulfilled, one point if the corresponding information was of insufficient detail and no points if the criterion was not fulfilled. We used the information if it met the methodological quality criteria. In addition, we assessed the risk of bias to guide sensitivity analyses and to explore the sources of heterogeneity.

### Statistical analysis

We selected hospital mortality as our primary outcome measure. If this outcome was not obtained, we preferentially used the outcomes in the following order: 28-day mortality and ICU mortality. The other outcome measure was the incidence rate of nosocomial infections, mortality at six months and the length of stay.

We analyzed data from the included studies using Review Manager (Review Manager, version 5.2). We calculated a pooled risk ratio for dichotomous data and mean differences for continuous data with 95% confidence intervals (CIs). The statistical heterogeneity of the data was explored and quantified by the Mantel-Haenszel chi-square test and the I^2^ test. Any obvious heterogeneity was predefined as *P* <*0*.05 with the Mantel-Haenszel chi-square test or an I^2^ >50%. A publication bias was assessed using funnel plot techniques.

### Subgroup meta-analyses

Subgroup meta-analyses were performed to determine the summary effect estimates of glutamine in specific patient populations (medical ICU, surgical ICU or mixed ICU), effects relative to a specific dosage (above 0.5 g/kg/day, between 0.3 g/kg/day and 0.5 g/kg/day, below 0.3 g/kg/day) and the effect of the mode of nutritional supplementation (parental nutrition, enteral nutrition or a combination of the two).

## Results

### Study location and selection

**Figure 1:**
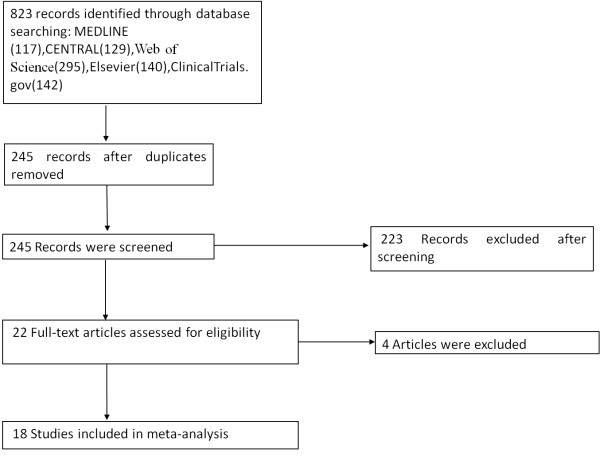
Flow diagram of the meta-analysis.

We identified a total 823 titles and abstracts after the primary search. Of these 823 items, 245 records remained after duplicates were removed. Based on their abstracts, 223 articles were determined to be non-relevant and were, therefore, excluded. The remaining 22 articles were retrieved for an eligibility assessment, four of which were deemed to be ineligible and were, therefore, excluded (Figure 1).

### Summary of studies

We included eighteen trials that compared glutamine supplementation with a placebo in ICU patients. Three trials were conducted in medical ICUs, eight in surgical ICUs and seven in mixed ICUs. High-dose glutamine (above 0.5 g/kg/day) was used in six trials, and four trails used glutamine at doses less than 0.3 g/kg /day; the other eight trails used glutamine at doses between 0.3 g/kg/day and 0.5 g/kg/day. Six studies used glutamine supplementation in which the patients were fed enterally, ten studies supplemented patients with glutamine by parental feeding, while the patients were fed using a combination of the two methods in the other two studies. An additional DOCX file shows this in more detail [see Additional file 2].

The overall description of the target population, a clear description of nosocomial infections, exclusion criteria, clinical condition and severity of the disease are summarized in Additional files [see Additional files 3, 4, 5].

### The impact on mortality

**Figure 2:**
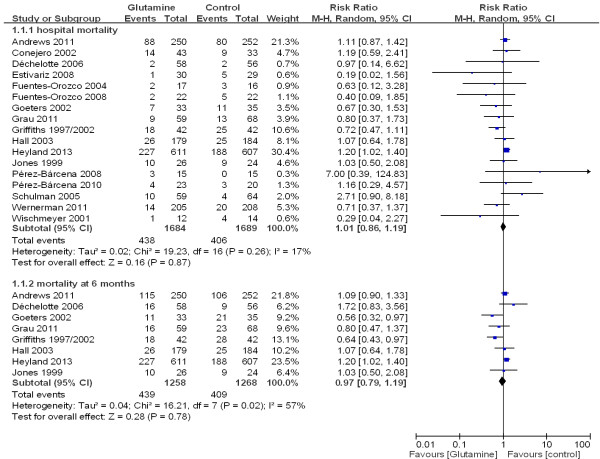
**The effect of glutamine supplementation on mortality in critically ill patients (fixed effects modes).** Gln: glutamine.

**Figure 3:**
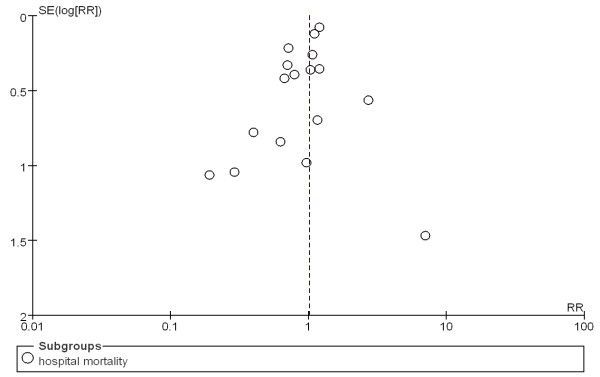
Funnel plot of the published studies in relation to the mortality meta-analysis (17 studies).

The overall effect of glutamine supplementation on the mortality rates was estimated from 17 trials, which included a total of 3,383 patients (Figure 2). We detected no evidence of a publication bias after a funnel plot analysis (Figure 3), and the heterogeneity was also determined to be non-significant (*P* = 0.26, I^2^ = 17%). Hospital mortality and six-month mortality were not significantly different between glutamine group and control group (RR 1.01; 95% CI, 0.86 to 1.19; *P* = 0.87; RR 0.97; 95% CI, 0.79 to 1.19; *P* = 0.78) (Figure 2).

### Subgroup analyses of specific patient populations

To determine the effect of glutamine on mortality in specific patient populations, we performed subgroup analyses of the trials according to whether the patients were in a medical ICU, a mixed ICU or a surgical ICU. There was a trend toward reduced mortality among patients who received glutamine as compared with patients who did not receive glutamine (10.8% versus14.4%; RR 0.77; 95% CI, 0.48 to 1.23), but this finding was not statistically significant (*P* = 0.27). In the medical ICU and mixed ICU subgroups, however, there was no significant difference in mortality between the glutamine group and the control group (see Additional file 2, Figure 4).

**Figure 4:**
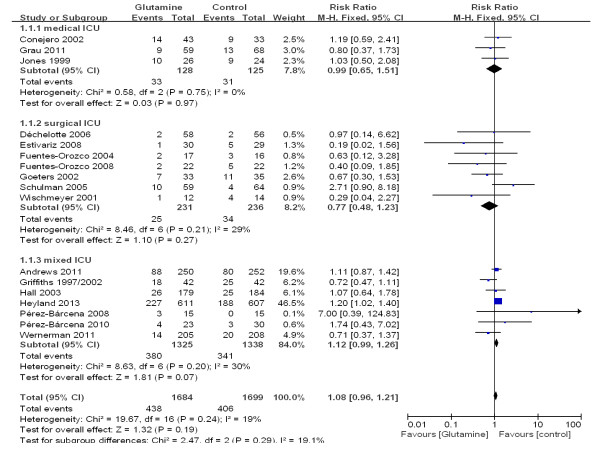
A subgroup meta-analysis of the effect of glutamine in specific patient populations on the mortality rate (fixed effects modes).

### Subgroup analyses of the modes of nutritional supplementation

To explore the effect of glutamine on mortality in ICU patients with different modes of nutritional supplementation, we evaluated subgroup analyses of the trials according to whether the patients were given enteral nutrition or parental nutrition. In three subgroups, there was no significant difference in mortality between patients who received glutamine and patients who did not receive glutamine (see Additional file 2, Figure 5).

**Figure 5:**
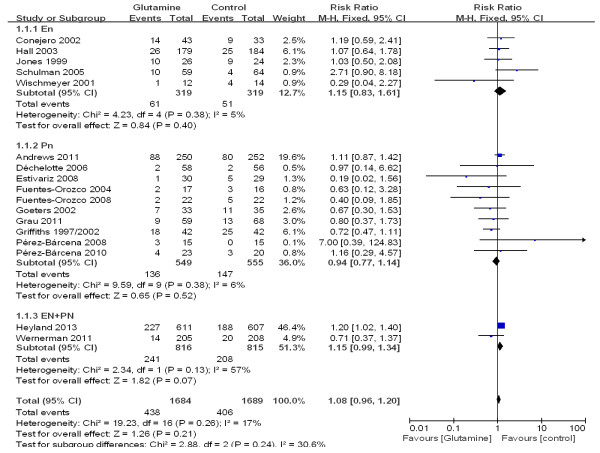
A subgroup meta-analysis of the effect of glutamine supplementation in different nutritional modes on the mortality rate in critically ill patients (fixed effects modes).

### A subgroup analysis of different glutamine dosages

To determine the effect on mortality of different dosages of glutamine that were given to ICU patients, we did a subgroup analysis of the trials according to glutamine dosages (above 0.5 g/kg/day, between 0.3 g/kg/day and 0.5 g/kg/day and below 0.3 g/kg/day). In the high dosage subgroup (above 0.5 g/kg/day), the mortality rate in the glutamine group was significantly higher than that of the control group (33.5% versus 28.2%; RR 1.18; 95% CI, 1.02 to 1.38; *P* = 0.03). In the other two subgroups, however, no difference was found in the treatment effect on mortality between the glutamine and control groups (see Additional file 2, Figure 6).

**Figure 6:**
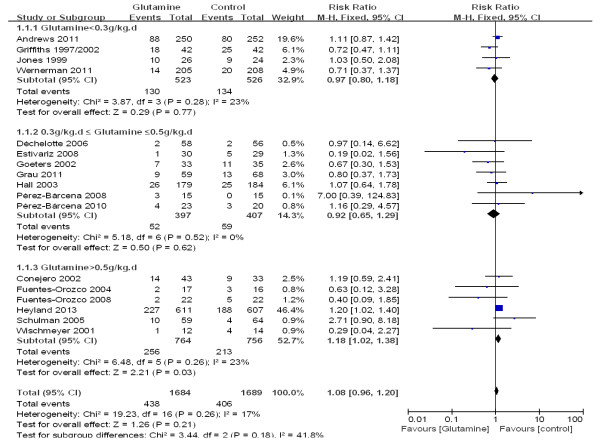
A subgroup meta-analysis of the different dosages of glutamine on mortality in critically ill patients (fixed effects modes).

### Impact on nosocomial infections

**Figure 7:**
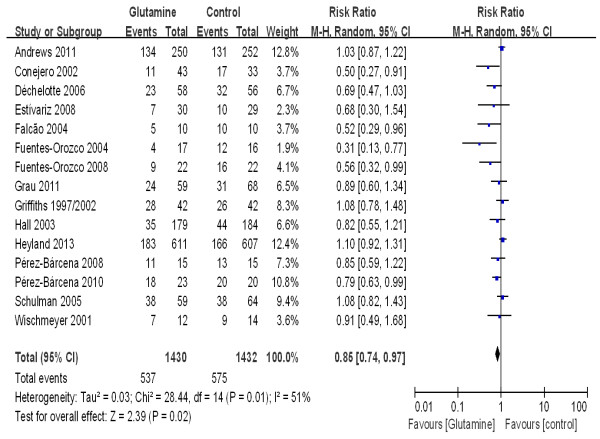
The effect of glutamine supplementation on the acquisition of new infections in critically ill patients (random effects modes).

**Figure 8:**
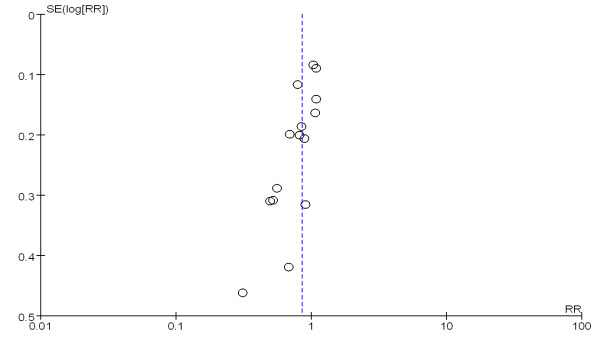
A funnel plot of the published studies in relation to the new infection meta analysis (15 studies).

**Figure 9:**
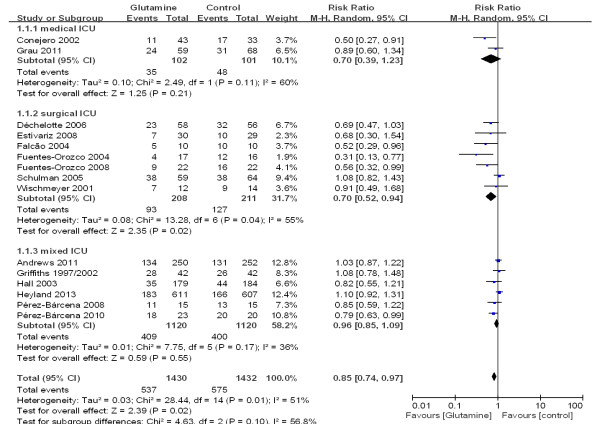
A subgroup meta-analysis of the effect of glutamine in specific patient populations on the acquisition of new infections in critically ill patients (random effects modes).

**Figure 10:**
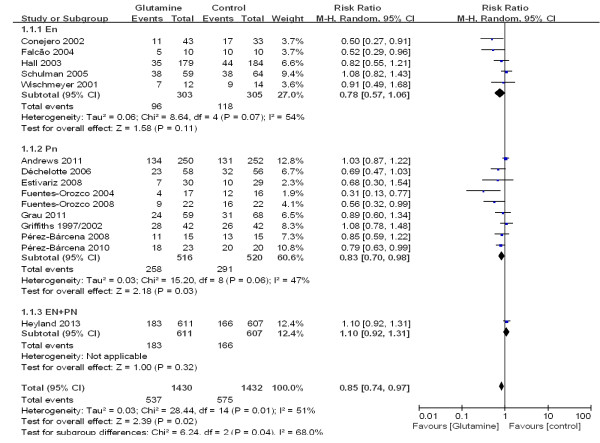
A subgroup meta-analysis of the effect of glutamine supplementation in different nutritional modes on the acquisition of new infections in critically ill patients (random effects modes).

To estimate the overall effect of glutamine supplementation on the incidence rate of nosocomial infections, 15 trials, which included 2,862 patients, were evaluated (Figure 7). No evidence of a publication bias was observed following a funnel plot assessment (Figure 8), but the heterogeneity was obvious (*P* = 0.01, I^2^ = 51%). The incidence of nosocomial infections in the glutamine group was significantly lower than that of the control group (RR 0.85; 95% CI, 0.74 to 0.97; *P* = 0.02). We performed a subgroup analysis of the trials according to specific patient populations (medical ICU, surgical ICU or mixed ICU). In the surgical ICU subgroup, glutamine supplementation statistically reduced the rate of nosocomial infections (44.7% versus 60.2%; RR 0.70; 95% CI, 0.52 to 0.94; *P* = 0.04). However, in the medical ICU and mixed ICU subgroups, no statistically significant difference was found between the glutamine group and the control group (Figure 9). Then we evaluated subgroup analyses of the trials according to mode of nutritional supplementation. In the parental nutrition subgroup, glutamine supplementation statistically reduced the rate of nosocomial infections (50.0% versus 55.9%; RR 0.83; 95% CI, 0.70 to 0.98; *P* = 0.03) (Figure 10).

### Impact on the length of stay

**Figure 11:**
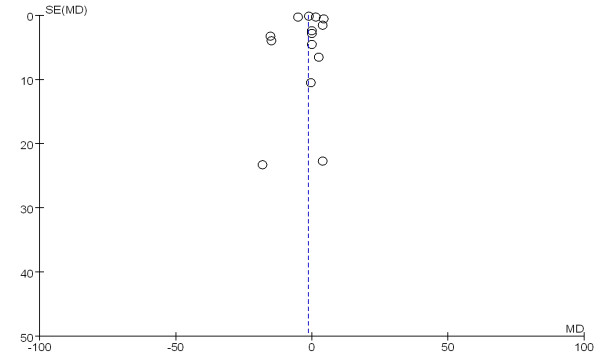
A funnel plot of published studies in relation to the hospital length of stay meta-analysis (14 studies).

**Figure 12:**
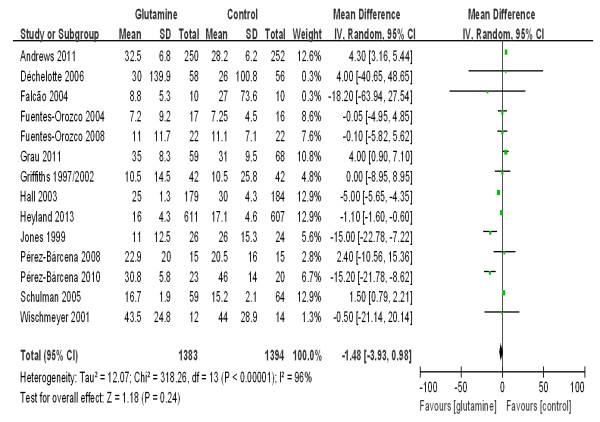
The effect of glutamine supplementation on the hospital length of stay in critically ill patients.

The patient length of hospital stay was reported in 14 trials that enrolled a total of 2,777 patients. We detected no evidence of a publication bias following a funnel plot assessment (Figure 11), but the heterogeneity was significantly different (*P* <0.00001, I^2^ = 96%). No difference was found between the groups with respect to the length of hospital stay (WMD, -1.48 days; 95% CI, -3.93 to 0.98; *P* = 0.24) (Figure 12).

## Discussion

Similar to previous meta-analyses [17], glutamine supplementation reduced nosocomial infections among critically ill patients. However, unlike previous meta-analyses [17], we found that glutamine supplementation conferred no overall mortality benefit in critically ill patients. Furthermore, our subgroup analyses suggested that high dosage glutamine supplementation (above 0.5 g/kg/day) significantly increased mortality in the observed critically ill patients. In addition, we did not observe a shortening of the length of hospital stay due to glutamine supplementation.

Glutamine depletion impairs gastrointestinal integrity and immunologic function and is an independent prognostic factor for poor outcomes in ICU patients [4]. Thus, the investigators of some studies that provided glutamine supplementation during critical illness expected to reduce nosocomial infection and, therefore, improve patient prognosis. In 1997, Griffiths *et al*. [18] showed that a glutamine-containing parental solution improved the patient six-month survival rate and reduced hospital costs in severely ill patients. Following that study, many future studies [11-13] suggested that supplementation of parental nutrition with glutamine decreased nosocomial infections following a critical illness. Additional studies [19-21] explored the prognostic role of the enteral administration of supplemental glutamine in critically ill patients; however, these findings were rather diverse and remain unclear. Therefore, it was suggested that, as a guideline [22], when parental nutrition is used in the ICU, consideration should be given to supplement it with glutamine. However, this viewpoint lacks the powerful evidence that is provided by larger trials.

Disappointingly, two larger trials showed no evidence of a benefit with glutamine as a nutritional supplement. The SIGNET study [15] was a randomized, double-blind, factorial, controlled trial that involved 502 ICU patients. Its result showed no effect on the rate of nosocomial infection incidence or on the rate of mortality when parenteral nutrition was supplemented with glutamine. The problem with the SIGNET study was giving a low dose of glutamine (20.2 g/day). The recently published REDOX study [14], the largest trial involving glutamine supplementation, suggested that glutamine supplementation was associated with an increase in mortality without any benefits for critically ill patients with multi-organ failure. However, there are problems with the REDOX study. It was unbalanced in patients with three or more organ failures and nutrition supplementation between glutamine and control group. Therefore, it was urgent to reevaluate the effect of glutamine in critically ill patients.

Many studies [4,23,24] and meta-analyses [17,25] of randomized trials suggest that nutritional glutamine supplementation in surgical critically ill patients may be associated with improved survival. Our subgroup meta-analyses suggested that the effect of glutamine supplementation differed by ICU setting. Patients in surgical ICUs benefited from glutamine supplementation, with the prior observation of a reduced nosocomial infection rate and a tendency for decreased mortality, in contrast to patients in medical ICUs and mixed ICUs. However, the exact mechanism of this benefit is unclear. One possible reason may be that surgical critically ill patients rely more on glutamine because their intestinal tracts may be impaired, and glutamine supplementation may be a primary means of obtaining glutamine during critical illness [26-28]. However, medical ICU patients [29] and some mixed ICU patients [30-32] can obtain glutamine from food in addition to glutamine supplementation. Therefore, enteral supplementation is only associated with a marginal effect on outcomes.

Reported studies [5,6] have suggested that both glutamine-supplemented parenteral nutrition and enteral nutrition may prevent bacterial translocation, but this effect may be different between the parenteral and enteral nutrition diets. We showed that the mortality rate of ICU patients was reduced when parenteral nutrition was supplemented with glutamine, but this supplementation did not provide a benefit when given via the gastrointestinal tract. A possible cause of this result is that those ICU patients with good intestinal function can maintain good nutrition without glutamine supplementation. Therefore, glutamine supplementation by parental feeding may be the primary method of obtaining glutamine during critical illness, because many of these patients are affected by gastrointestinal dysfunction. Furthermore, while the dosage of glutamine added by the enteral route not enough to produce a sufficient effect, it had no a favorable impact on outcome by its influence on intestinal epithelium and maintenance of gut integrity [22].

Lower plasma glutamine levels have been associated with a higher mortality rate in critically ill patients [4]; however, critical illness is not necessarily associated with a low plasma glutamine [33]. Five RCTs [11,14,18,19,26] in our meta-analysis examined the plasma glutamine level; patients presented with a low baseline glutamine level (<420 μmol/L) in only two of these RCTs [18,19]. Patients with a normal plasma glutamine level cannot benefit from glutamine supplementation. Rodas *et al*. [34] discovered that elevated baseline levels of glutamine in the plasma (a value of >930 μmol/L) of critically ill patients was actually associated with increased mortality. Thus, high dosage glutamine supplementation caused a harmful effect, such as high urea levels [15], instead of resulting in a benefit. Our meta-analysis showed that glutamine supplementation at a dosage higher than 0.5 g/kg/day increased mortality in ICU patients, while ICU patients may only obtain a benefit from glutamine at a dosage of between 0.3 g/kg/day and 0.5 g/kg/day. Glutamine supplementation at a higher dosage was used in the REDOX study, which may account for its disadvantageous role. It is, therefore, urgent to choose an optimal dose of glutamine, given the discrepancy among different studies. To solve the problem, we can monitor glutamine plasma concentration before giving glutamine to critically ill patients. It is suggested that ICU patients be given glutamine at a dosage of between 0.3 g/kg/day and 0.5 g/kg/day when they present with a low baseline glutamine level (<420 μmol/L). Appropriate glutamine plasma concentration would be a treatment target of glutamine supplementation.

Some limitations of our analysis should be noted. First, we were unable to include all relevant studies because our meta-analyses could only take into account sources written in English. In addition, some published trials only reported the median and range. Using formulas, we estimated the mean and variance of the length of stay from the median, range and the size of the trial.

## Conclusions

Similar to a previous meta-analysis [17], glutamine supplementation reduced nosocomial infections among critically ill patients. However, unlike previous meta-analyses [17], we found that glutamine supplementation conferred no overall mortality benefit in critically ill patients. Administration of glutamine to surgical ICU patients resulted in a significant reduction of infectious complications and may reduce mortality in these patients, which is in line with previous meta-analyses. Furthermore, our subgroup analyses suggested that high dosage glutamine supplementation (above 0.5 g/kg/day) significantly increased mortality in the observed critically ill patients. In addition, we did not observe a shortening of the length of hospital stay due to glutamine supplementation. The discrepancies between the REDOX study and earlier evidence syntheses may be due to limitations of previous trials. However, the REDOX study is also problematic. Appropriate glutamine plasma concentration by monitoring might be a treatment target of glutamine supplementation. The effectiveness of glutamine supplementation in critically ill patients remains uncertain. Results from additional large-scale, high-quality RCTs are needed.

## Key messages

• The effects of glutamine supplementation on mortality differed according to patient populations, modes of nutrition and glutamine dosages.

• Glutamine supplementation conferred no overall mortality benefit among critically ill patients.

• Glutamine supplementation reduced nosocomial infections among critically ill patients.

• Glutamine supplementation did not reduce the length of stay among critically ill patients.

• Surgical patients benefited from glutamine supplementation.

## Abbreviations

MICU: medical intensive care unit; REDOX study: REducing Deaths due to OXidative Stress; SICU: surgical intensive care unit; SIGNET study: Scottish Intensive care Glutamine or seleNium Evaluative Trial; Pn: Parental nutrition; En: Enteral nutrition; WMD: Weighted Mean Difference.

## Competing interests

The authors declare that they have no competing interests.

## Authors’ contributions

QC carried out the acquisition, analysis and interpretation of data and participated in drafting, editing and submitting the manuscript. YY contributed to the design and coordination of the study. HH was responsible for selecting studies. JX and SC were two reviewers screening titles and abstracts to determine whether a particular study met the inclusion criteria. AL contributed to the design, as well as the acquisition, analysis and interpretation of data. HW contributed to study analysis and interpretation of data, and HQ was responsible for conception and design, and revising the manuscript for important intellectual content. All authors read and approved the final manuscript.

## Supplementary Material

Additional file 1 Search strategy and excluded references: the file includes electronic database search strategy and all excluded full-text articles. Click here for file

Additional file 2 Summary of the studies included in the meta-analysis: this file contains a table of authors, population types, nutritional modes, glutamine dose, Jadad score and outcomes of included studies. Click here for file

Additional file 3 Summary of the population included in the meta-analysis: this file contains diagnosis and comorbidities of included studies. Click here for file

Additional file 4 Summary of the population included in the meta-analysis: this file contains a table of exclusion criteria and the definition of nosocomial infection of included studies. Click here for file

Additional file 5 Summary of the population included in the meta-analysis: this file contains a table of comorbidities, SOFA score, glutamine plasma concentration and time to add nutrition of included studies. Click here for file
