# Correction: The effect of glutamine therapy on outcomes in critically ill patients: a meta-analysis of randomized controlled trials

**DOI:** 10.1186/cc13926

**Published:** 2014-06-17

**Authors:** Qi-Hong Chen, Yi Yang, Hong-Li He, Jian-Feng Xie, Shi-Xia Cai, Ai-Ran Liu, Hua-Ling Wang, Hai-Bo Qiu

**Affiliations:** 1 Department of Critical Care Medicine, Zhong-Da Hospital, School of Medicine, Southeast University, 87 Dingjiaqiao Road, Nanjing 210009, P.R. China; 2 Department of Critical Care Medicine, Su-Bei Hospital of Jiangsu Provience & Clinical Medical School, Yangzhou University, Yangzhou, Jiangsu, P.R. China

## 

After publication of our article in a recent issue of *Critical Care*[1], inconsistencies were identified in Figure 6. Four trials - by Fuentes-Orozco and colleagues [2,3], Hall and colleagues [4], and Goeters and colleagues [5] - were allocated to the wrong subgroup in our meta-analysis. They should have been included in the first subgroup (glutamine <0.3 g/kg per day); this does not change the significance of the results (*P* = 0.01). The correct Figure 6 is given here in full as Figure 1.

**Figure 1:**
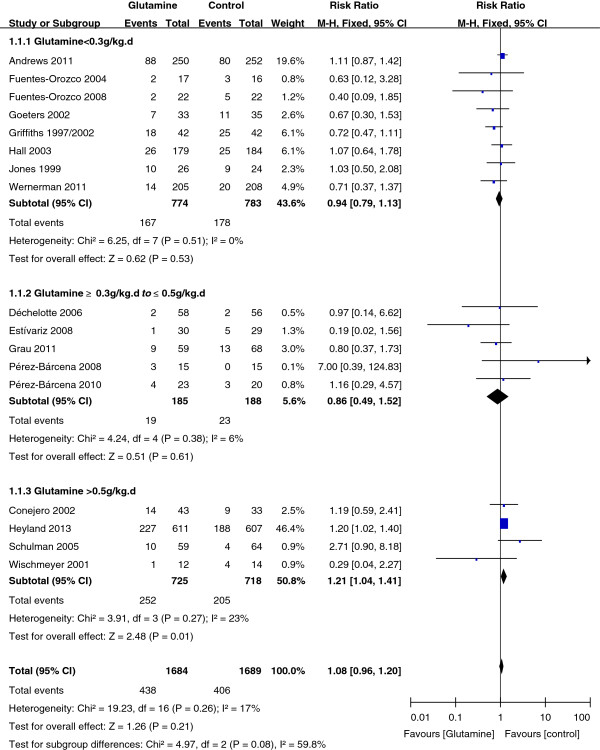
**A subgroup meta-analysis of the effects of different dosages of glutamine on mortality in critically ill patients (fixed effects models).** CI, confidence interval; M-H, Mantel-Haenszel.

## Competing interests

The authors declare that they have no competing interests.
