# Supplementary material for: The effect of glutamine therapy on outcomes in critically ill patients: a meta-analysis of randomized controlled trials
Source: Crit Care. 2014 Jan 9;18(1):R8. doi: 10.1186/cc13185 (PMC4057299; doi:10.1186/cc13185)
Supplement: Additional file 1 — Search strategy and excluded references: the file includes electronic database search strategy and all excluded full-text articles. [file cc13185-S1.docx]

**Additional file1. Search strategy and excluded references**

**Search strategy**

Database: Ovid MEDLINE(R) In-Process & Other Non-Indexed Citations and Ovid MEDLINE(R) <1948 to Present>

------------------------------------------------------------------------

## 1 (“glutamine” or “glutamine dipeptides” or “L –glutamine” or “glutamine supplementation”) and (“critical care” or “critical patients” or “critical ill” or “critically ill patients” or “critical illness” or “serious illness” or “seriously ill” or “intensive care units” or “intensive care” or “surgical intensive care unit” or “SICU” or “critical care medicine”)/ (668)

2     limit 1 to humans (545)

3 limit 2 to Clinical Trial (117)

Database: The Cochrane Controlled Trials Register

--------------------------------------------------------------------------------

1  (**“glutamine” or “glutamine dipeptides” or “L –glutamine” or “glutamine supplementation”) and (“critical care” or “critical patients” or “critically ill patients” or “critical illness” or “serious illness” or “seriously ill” or “intensive care units” or “intensive care” or “surgical intensive care unit” or“SICU” or “critical care medicine”**). (144)

2  limit 1 to humans and clinical trial (129)

Database: Web of Science(SCI/SSCI/AHCI/CPCI)

--------------------------------------------------------------------------------

1  (**“glutamine” or “glutamine dipeptides” or “L –glutamine” or “glutamine supplementation”) and (“critical care” or “critical patients” or“critical ill”or “critical illness” or “serious illness” or “seriously ill” or “intensive care units” or “intensive care” or “surgical intensive care unit” or“SICU” or “critical care medicine”**). (386)

2  limit 1 to humans and clinical trial (295)

Database: Elsevier

--------------------------------------------------------------------------------

1  (**“glutamine” or “glutamine dipeptides” or “L –glutamine” or “glutamine supplementation”) and (“critical care” or “critical patients” or“critical ill”or “critical illness” or “serious illness” or “seriously ill” or “intensive care units” or “intensive care” or “surgical intensive care unit” or“SICU” or “critical care medicine”**). (557)

2  limit 1 to humans and clinical trial (140)

Database: ClinicalTrials.gov

--------------------------------------------------------------------------------

(**“glutamine” or “**glutamine **d**ipeptides**” or “**L **–**glutamine**” or “**glutamine supplementation**”) and (“critical care” or “critical patients” or“critical ill”or “critical illness” or “serious illness” or “seriously ill” or “intensive care units” or “intensive care” or “surgical intensive care unit” or“SICU” or “critical care medicine”**) (142)

**Excluded articles**

1、[Griffiths RD](http://www.ncbi.nlm.nih.gov/pubmed?term=Griffiths%20RD%5BAuthor%5D&cauthor=true&cauthor_uid=12093428), [Allen KD](http://www.ncbi.nlm.nih.gov/pubmed?term=Allen%20KD%5BAuthor%5D&cauthor=true&cauthor_uid=12093428), [Andrews FJ](http://www.ncbi.nlm.nih.gov/pubmed?term=Andrews%20FJ%5BAuthor%5D&cauthor=true&cauthor_uid=12093428)，et al. Infection, multiple organ failure, and survival

in the intensive care unit: influence of glutamine-supplemented parenteral nutrition on

acquired infection. [Nutrition.](http://www.ncbi.nlm.nih.gov/pubmed/12093428) 2002 Jul-Aug;18(7-8):546-52

2．[Schulman AS](http://www.ncbi.nlm.nih.gov/pubmed?term=Schulman%20AS%5BAuthor%5D&cauthor=true&cauthor_uid=16509783), [Willcutts KF](http://www.ncbi.nlm.nih.gov/pubmed?term=Willcutts%20KF%5BAuthor%5D&cauthor=true&cauthor_uid=16509783), [Claridge JA](http://www.ncbi.nlm.nih.gov/pubmed?term=Claridge%20JA%5BAuthor%5D&cauthor=true&cauthor_uid=16509783)，et al. Does enteral glutamine supplementation decrease infectious morbidity? [Surg Infect (Larchmt).](http://www.ncbi.nlm.nih.gov/pubmed/16509783) 2006 Feb;7(1):29-35

3．[Beale RJ](http://www.ncbi.nlm.nih.gov/pubmed?term=Beale%20RJ%5BAuthor%5D&cauthor=true&cauthor_uid=18007263), [Sherry T](http://www.ncbi.nlm.nih.gov/pubmed?term=Sherry%20T%5BAuthor%5D&cauthor=true&cauthor_uid=18007263), [Lei K](http://www.ncbi.nlm.nih.gov/pubmed?term=Lei%20K%5BAuthor%5D&cauthor=true&cauthor_uid=18007263), et al. Early enteral supplementation with key pharmaconutrients improves Sequential Organ Failure Assessment score in critically ill patients with sepsis: outcome of a randomized, controlled, double-blind trial. [Crit Care Med.](http://www.ncbi.nlm.nih.gov/pubmed/?term=Early+enteral+supplementation+with+key+pharmaconutrients) 2008 Jan;36(1):131-44

4. [Sodergren MH](http://www.ncbi.nlm.nih.gov/pubmed?term=Sodergren%20MH%5BAuthor%5D&cauthor=true&cauthor_uid=21044933), [Jethwa P](http://www.ncbi.nlm.nih.gov/pubmed?term=Jethwa%20P%5BAuthor%5D&cauthor=true&cauthor_uid=21044933), [Kumar S](http://www.ncbi.nlm.nih.gov/pubmed?term=Kumar%20S%5BAuthor%5D&cauthor=true&cauthor_uid=21044933),et al. Immunonutrition in patients undergoing major upper gastrointestinal surgery: a prospective double-blind randomised controlled study. [Scand J Surg.](http://www.ncbi.nlm.nih.gov/pubmed/?term=c+urrent+studies+suggest+immunonutrition+decreases+the+inflammatory) 2010;99(3):153-61
