# Supplementary material for: The effect of glutamine therapy on outcomes in critically ill patients: a meta-analysis of randomized controlled trials
Source: Crit Care. 2014 Jan 9;18(1):R8. doi: 10.1186/cc13185 (PMC4057299; doi:10.1186/cc13185)
Supplement: Additional file 2 — Summary of the studies included in the meta-analysis: this file contains a table of authors, population types, nutritional modes, glutamine dose, Jadad score and outcomes of included studies. [file cc13185-S2.docx]

| **Author** | **year** | **population** | **APACHE II** | | **Jadad score** | **Nutri-tion** | **Gln dose** | **MV**  **(events**  **/total)** | **hospital mortality** | | **mortality at 6 month** | | **Infectious morbidity** | | **Hospital length of stay** | |
| --- | --- | --- | --- | --- | --- | --- | --- | --- | --- | --- | --- | --- | --- | --- | --- | --- |
|  |  |  | **Gln** | **Con** |  | **type** | **g/kg.d** |  | **Gln** | **Con** | **Gln** | **Con** | **Gln** | **Con** | **Gln** | **Con** |
| Griffiths18 | 1997 | Mixed ICU | 18 | 17 | 5 | PN | 0.26 | 78/84 | 18/42 | 25/42 | 18/42 | 28/42 | 28/42 | 26/42 | 10.5±14.5 | 10.5±25.8^a^ |
|  |  |  | (11-14) | (11-31) |  |  |  |  |  |  |  |  |  |  |  |  |
| Jones 19 | 1999 | Medical ICU | 17.5 | 16.5 | 3 | EN | 0.25 | 42/50 | 10/26 | 9/24 | 12/26 | 10/24 | NA | NA | 11.0±12.5 | 26.0±15.3^a^ |
|  |  |  | (11-37) | (12-24) |  |  |  |  |  |  |  |  |  |  |  |  |
| Wischmeyer21 | 2001 | Surgical ICU | NA | NA | 5 | EN | 0.57 | 18/26 | 1/12 | 4/14 | NA | NA | 7/12 | 9/14 | 43.5±24.8 | 44.0±28.9^a^ |
| Conejero20 | 2002 | Medical ICU | 20 | 18 | 5 | EN | 0.5-0.6 | 60/76 | 12/43 | 9/33 | NA | NA | 11/43 | 17/33 | NA | NA |
|  |  |  | (6-34) | (6-46) |  |  |  |  |  |  |  |  |  |  |  |  |
| Goeters26 | 2002 | Surgical ICU | NA | NA | 3 | PN | 0.2 | NA | 7/33 | 11/35 | 11/33 | 21/35 | NA | NA | 46.0+49.1 | 39.4±31.3^a^ |
| Hall27 | 2003 | Mixed ICU | 14 | 14 | 3 | EN | 0.29 | NA | 26/179 | 25/184 | 27/179 | 30/184 | 35/179 | 44/184 | 25.0±1.3 | 30.0+4.3^a^ |
|  |  |  | (9–18) | (10–18) |  |  |  |  |  |  |  |  |  |  |  |  |
| Falcão28 | 2004 | Surgical ICU | NA | NA | 2 | EN | 0.43 | NA | NA | NA | NA | NA | 5/10 | 10/10 | 8.8±5.3 | 27.0±73.6^a^ |
| Fuentes-O25 | 2004 | Surgical ICU | NA | NA | 2 | PN | 0.27 | 33/33 | 2/17 | 3/16 | NA | NA | 4/17 | 12/16 | 7.1±9.2 | 7.3±4.5 |
| Schulman24 | 2005 | Surgical ICU | 18.6 (0.9) | 17.9(0.9) | 3 | EN | 0.6 | NA | 10/59 | 6/64 | NA | NA | 38/59 | 38/64 | 16.7±1.9 | 15.2±2.1 |
| Déchelotte29 | 2006 | Surgical ICU | NA | NA | 5 | PN | 0.34 | NA | 2/58 | 2/56 | 16/58 | 9/56 | 23/58/ | 32/56 | 30.0±139.9 | 26.0±100.8^a^ |
| Estívariz11 | 2008 | Surgical ICU | 13.4(1.4) | 13.1(1.2) | 5 | PN | 0.34 | 39/59 | 1/30 | 5/29 | NA | NA | 7/30 | 10/29 | NA | NA |
| Pérez-Bárcena | 2008 | Mixed ICU | 10.3(1.6) | 10.7(1.9) | 4 | PN | 0.34 | 30/30 | 3/15 | 0/15 | NA | NA | 11/15 | 3/15 | 22.9±20.0 | 20.5±16.0 |
| Fuentes-O13 | 2008 | Surgical ICU | 10.3(1.6) | 10.7(1.9) | 4 | PN | 0.27 | 44/44 | 2/22 | 5/22 | NA | NA | 9/22 | 16/22 | 11.0±11.7 | 11.1±7.1 |
| Pérez-Bárcena  30 | 2010 | Mixed ICU | 19.2(3.2) | 15.1(9.3) | 3 | PN | 0.35 | 45/45 | 4/23 | 3/20 | NA | NA | 18/23 | 20/20 | 30.8±5.8 | 46.0±14.0^a^ |
| Wernerman31 | 2011 | Mixed ICU | 21 | 22 | 5 | PN+  EN | 0.28 | NA | 14/205 | 20/208 | NA | NA | NA | NA | NA | NA |
| Andrews15 | 2011 | Mixed ICU | 21 (16–25) | 20 (15–25) | 5 | PN | 0.29 | NA | 88/250 | 80/252 | 115/250 | 106/252 | 134/250 | 131/252 | 32.5±6.8 | 28.2±6.2^a^ |
| Grau32 | 2011 | Medical ICU | 19 (15–21) | 18 (15–23) | 4 | PN | 0.5 | 31/121 | 9/59 | 13/68 | 16/59 | 23/68 | 24/59 | 31/68 | 35.0±8.3 | 31.0±9.5^a^ |
| Heyland14 | 2013 | Mixed ICU | 26.6(7.6) | 26(7.4) | 5 | EN+  PN | 0.35 | 134/1217 | 227/611 | 188/607 | 259/611 | 218/607 | 183/611 | 166/607 | 16.0±4.3 | 17.1±4.6^a^ |

**Additional file2. Summary of the studies included in the meta-analysis**

PN: parental nutrition; EN: enteral nutrition; Gln: glutamine; Con: control; NA: not available

^a^ Calculated val
