# Supplementary material for: The effect of glutamine therapy on outcomes in critically ill patients: a meta-analysis of randomized controlled trials
Source: Crit Care. 2014 Jan 9;18(1):R8. doi: 10.1186/cc13185 (PMC4057299; doi:10.1186/cc13185)
Supplement: Additional file 3 — Summary of the population included in the meta-analysis: this file contains diagnosis and comorbidities of included studies. [file cc13185-S3.docx]

| **study** | **diagnosis** | | | | | | | | | **co morbidities** | | | |
| --- | --- | --- | --- | --- | --- | --- | --- | --- | --- | --- | --- | --- | --- |
|  | **sepsis** | **respiratory** | **Cardio-** | **neurology** | **surgical** | **trauma** | **Pancreatitis** | | **others** | **Diabetes** | **myocardial** | **stroke** | **COPD** |
|  |  |  | **vascular** |  |  |  |  |  |  |  | **infarction** |  |  |
| Griffiths | 37 | 24 | 8 | 0 | 0 | 5 | 7 |  | 3 | NA |  |  |  |
| 1997/2002 |  |  |  |  |  |  |  |  |  |  |  |  |  |
| [Jones](http://www.ncbi.nlm.nih.gov/pubmed?term=Jones%20C%5BAuthor%5D&cauthor=true&cauthor_uid=9990574) | 4 | 32 | 2 | 0 | 0 | 6 | 0 |  | 6 | NA |  |  |  |
| 1999 |  |  |  |  |  |  |  |  |  |  |  |  |  |
| Conejero | 48 | 37 | 25 | 0 | 7 | 11 | 0 |  | 13 | NA |  |  |  |
| 2002 |  |  |  |  |  |  |  |  |  |  |  |  |  |
| Hall | 20 | 54 | 25 | 72 | 16 | 154 | 0 |  | 22 | 26/363 | 17/363 | 11/363 | 31/363 |
| 2003 |  |  |  |  |  |  |  |  |  |  |  |  |  |
| Falcão | NA |  |  |  |  |  |  |  |  | NA |  |  |  |
| 2004 |  |  |  |  |  |  |  |  |  |  |  |  |  |
| Fuentes-O | NA |  |  |  |  |  |  |  |  | NA |  |  |  |
| 2004 |  |  |  |  |  |  |  |  |  |  |  |  |  |
| Schulman | 0 | 0 | 0 | 34 | 98 | 116 | 0 | 0 | 0 | NA |  |  |  |
| 2005 |  |  |  |  |  |  |  |  |  |  |  |  |  |
| Déchelotte | 0 | 0 | 0 | 0 | 65 | 38 | 11 | 0 | 0 | NA |  |  |  |
| 2006 |  |  |  |  |  |  |  |  |  |  |  |  |  |
| Estívariz | 0 | 0 | 0 | 0 | 59 | 0 | 32 | 0 | 0 | NA |  |  |  |
| 2008 |  |  |  |  |  |  |  |  |  |  |  |  |  |
| Pérez-Bárcena | | 6 |  |  | 8 | 16 |  |  |  | NA |  |  |  |
| 2008 |  |  |  |  |  |  |  |  |  |  |  |  |  |
| **study** | **diagnosis** | | | | | | | | **co morbidities** | | | | |
|  | **sepsis** | **respiratory** | **Cardio-** | **neurology** | **surgical** | **trauma** | **Pancreatitis** | **others** | **Diabetes** | **myocardial** | **stroke** | **COPD** |  |
|  |  |  | **vascular** |  |  |  |  |  |  | **infarction** |  |  |  |
| Pérez-Bárcena | | NA |  |  |  |  |  |  | NA |  |  |  |  |
| 2010 |  |  |  |  |  |  |  |  |  |  |  |  |  |
| Andrews | 282 | 0 | 0 | 0 | 0 | 0 | 0 | 220 | NA |  |  |  |  |
| 2011 |  |  |  |  |  |  |  |  |  |  |  |  |  |
| Grau | 35 | 0 | 3 | 0 | 44 | 31 | 0 | 14 | NA |  |  |  |  |
| 2011 |  |  |  |  |  |  |  |  |  |  |  |  |  |
| [Wernerman](http://www.ncbi.nlm.nih.gov/pubmed?term=Wernerman%20J%5BAuthor%5D&cauthor=true&cauthor_uid=21658010) | 0 | 0 | 0 | 0 | 243 | 0 | 0 | 170 | NA |  |  |  |  |
| 2011 |  |  |  |  |  |  |  |  |  |  |  |  |  |
| [Goeters](http://www.ncbi.nlm.nih.gov/pubmed?term=Goeters%20C%5BAuthor%5D&cauthor=true&cauthor_uid=12352037) | 26 | 0 | 0 | 0 | 61 | 20 | 0 | 26 | NA |  |  |  |  |
| 2002 |  |  |  |  |  |  |  |  |  |  |  |  |  |
| [Wischmeyer](http://www.ncbi.nlm.nih.gov/pubmed?term=Wischmeyer%20PE%5BAuthor%5D&cauthor=true&cauthor_uid=11700398) | 0 | 0 | 0 | 0 | 0 | 0 | 0 | 220 | NA |  |  |  |  |
| 2001 |  |  |  |  |  |  |  |  |  |  |  |  |  |
| Heyland | 174 | 198 | 124 | 0 | 0 | 19 | 0 | 86 | NA |  |  |  |  |
| 2013 |  |  |  |  |  |  |  |  |  |  |  |  |  |

**Additional file3. Summary of the population included in the meta-analysis: This file contains diagnosis and comorbidities of included studies.**

NA: Not available
