# Supplementary material for: The effect of glutamine therapy on outcomes in critically ill patients: a meta-analysis of randomized controlled trials
Source: Crit Care. 2014 Jan 9;18(1):R8. doi: 10.1186/cc13185 (PMC4057299; doi:10.1186/cc13185)
Supplement: Additional file 4 — Summary of the population included in the meta-analysis: this file contains a table of exclusion criteria and the definition of nosocomial infection of included studies. [file cc13185-S4.docx]

| **study** | **Exclusion criteria** | **nosocomial infection is defined** |
| --- | --- | --- |
| Griffiths | age under 16 y; pregnancy; clinical | NA |
| 1997/2002 | severe liver failure; or malignant disease |  |
| [Jones](http://www.ncbi.nlm.nih.gov/pubmed?term=Jones%20C%5BAuthor%5D&cauthor=true&cauthor_uid=9990574) | age under 16 y; pregnancy; clinical | NA |
| 1999 | severe liver failure; or malignant disease |  |
| Conejero | age younger than 18 y, pregnancy, | CDC |
| 2002 | previous cardiopulmonary resuscitation, severe |  |
|  | alnutrition, diabetes, |  |
| Hall | renal failure, liver failure , cancer, infection with HIV, and previous use of | NA |
| 2003 | steroids, salicylates, other anti-inflammatory drugs, and |  |
|  | immunosuppressive drugs. |  |
| Falcão | COPD;renal failure; pregnancy;morbid obesity;severe brain injury | CDC |
| 2004 |  |  |
| Fuentes-O | renal failure;hepatic failure;severe neutropenia;receiving cytotoxic, |  |
| 2004 | radiation and/or steroid |  |
|  | therapy;hemodynamic instability |  |
| Schulman | tolerate oral feedings, be | CDC |
| 2005 | transferred out of the STICU, or die within 72 |  |
|  | hrs of admission |  |

| **study** | **Exclusion criteria** | **nosocomial infection is defined** |
| --- | --- | --- |
| Déchelotte | malnutrition or severe obesity;pregnancy or lactation,  hemodynamic failure;renal insufficiency;hepatic failure; severe or | CDC |
| 2006 | uncontrolled sepsis, persistent metabolic |  |
|  | acidosis, hypertriglyceridemia, immunosuppressive therapy or |  |
|  | constitutive immune deficiency |  |
| Estívariz  2008 | uncontrolled infection or history of active malignancy, | CDC |
|  | significant hepatic dysfunction |  |
| Pérez-Bárcena | ;allergic to glutamine;basic pathology included any serious; |  |
| 2008 | immune system condition;pregnancy;received |  |
|  | corticoids |  |
| Fuentes-O | renal failure;hepatic failure; severe neutropenia; | CDC |
| 2008 | receiving cytotoxic, radiation |  |
|  | or steroid therapy, and patients with hemodynamic instability |  |
| Pérez-Bárcena | allergic to glutamine, whose basic pathology included any |  |
| 2010 | serious immune system condition received corticoids |  |
|  | or any other immunosuppressant medication;pregnancy |  |
| Andrews | Pregnant women and people whose expected stay in the | CDC |
| 2011 | UK was <6 months |  |

| **study** | **Exclusion criteria** | **nosocomial infection is defined** |
| --- | --- | --- |
| Grau | receiving renal replacement therapy |  |
| 2011 | malnutrition; obesity;unstable hemodynamic status;unstable  hemodynamic status;hepatic disorders; chronic maintenance systemic steroid therapy;pregnant | CDC |
| [Wernerman](http://www.ncbi.nlm.nih.gov/pubmed?term=Wernerman%20J%5BAuthor%5D&cauthor=true&cauthor_uid=21658010) | patients received glutamine supplementation before screening; | CDC |
| 2011 | readmitted patients,who had previously participated |  |
|  | in the study; when informed consent was not received. |  |
| [Goeters](http://www.ncbi.nlm.nih.gov/pubmed?term=Goeters%20C%5BAuthor%5D&cauthor=true&cauthor_uid=12352037)  2002 | inborn disorders of amino acid metabolism | NA |
|  | discharge or death before completing study protocol |  |
|  | for 5 days |  |
| [Wischmeyer](http://www.ncbi.nlm.nih.gov/pubmed?term=Wischmeyer%20PE%5BAuthor%5D&cauthor=true&cauthor_uid=11700398) | severe hepatic or renal disease, pregnancy, death within | NA |
| 2001 | 72 hrs of admission; |  |
| Heyland | Absolute contraindication to enteral nutrients; severe | NA |
| 2013 | acquired brain injury ;Seizure Metastatic cancer |  |
|  | or Stage IV Lymphoma;Routine elective |  |
|  | cardiacdisorder surgery; Weight less than 50 kg or |  |
|  | greater than 200 kg ;burns |  |

**Additional file4. Summary of the population included in the meta-analysis**

CDC: Centers for Disease Control; NA: Not available
