# Supplementary material for: The effect of glutamine therapy on outcomes in critically ill patients: a meta-analysis of randomized controlled trials
Source: Crit Care. 2014 Jan 9;18(1):R8. doi: 10.1186/cc13185 (PMC4057299; doi:10.1186/cc13185)
Supplement: Additional file 5 — Summary of the population included in the meta-analysis: this file contains a table of comorbidities, SOFA score, glutamine plasma concentration and time to add nutrition of included studies. [file cc13185-S5.docx]

| **study** | **SOFA** | | **CRRT** | **Gln concentration**  **(mmol/l)** | | **Time to add nutrition** | **Comorbidities** | | | | | | |
| --- | --- | --- | --- | --- | --- | --- | --- | --- | --- | --- | --- | --- | --- |
|  | **Gln** | **Con** |  |  |  |  | **renal** | **diarrhea** | **abdominal** | **gastric** | **Hypertrigly** | **Hypergly** | **liver** |
|  |  |  |  | **Gln** | **Con** |  | **failure** |  | **distention** | **residuals** | **ceridemia** | **cemia** | **disorder** |
| Griffiths 1997/2002 | NA |  | 14/84 | 0.4 | 0.33 | intolerance of enteral feeding over 48 h or | NA |  |  |  |  |  |  |
|  |  |  |  |  |  | the presence of contraindications to enteral nutrition |  |  |  |  |  |  |  |
| [Jones 1999](http://www.ncbi.nlm.nih.gov/pubmed?term=Jones%20C%5BAuthor%5D&cauthor=true&cauthor_uid=9990574) | NA |  | 1/50 | 0.39 | 0.35 | Enteral nutrition was the normal route for | NA |  |  |  |  |  |  |
|  |  |  |  |  |  | nutrition on the ICU and usually started at about 48 h |  |  |  |  |  |  |  |
|  |  |  |  |  |  | postadmission |  |  |  |  |  |  |  |
| Conejero 2002 | NA |  | 13/76 | NA |  | NA |  | 6 | 5 | 20 |  |  |  |
| Hall 2003 | NA |  | NA | NA |  | was commenced as soon as practicable and when | 38 | 71 |  |  |  |  |  |
|  |  |  |  |  |  | the patients were haemodynamically stable |  |  |  |  |  |  |  |

| **study** | **SOFA** | | **CRRT** | **Gln**  **(mmol/l)** | | **Time to add nutrition** | **Comorbidities** | | | | | | |
| --- | --- | --- | --- | --- | --- | --- | --- | --- | --- | --- | --- | --- | --- |
|  | **Gln** | **Con** |  |  |  |  | **renal** | **diarrhea** | **abdominal** | **gastric** | **Hypertrigly** | **Hypergly** | **liver** |
|  |  |  |  | **Gln** | **Con** |  | **failure** |  | **distention** | **residuals** | **ceridemia** | **cemia** | **disorder** |
| Falcão 2004 | NA |  | NA | NA |  | no later than 48 h after admission |  |  |  |  |  |  |  |
| Fuentes-Orozco 2004 | NA |  | NA | NA |  | commenced the morning after surgery | NA |  |  |  |  |  |  |
| Schulman 2005 | NA |  | NA | NA |  | NA |  |  |  |  |  |  |  |
| Déchelotte 2006 | NA |  | NA | NA |  | NA | 13 |  |  |  | 18 | 50 | 57 |
| Estívariz 2008 | NA |  | NA | 0.432 | 0.405 | NA |  |  |  |  |  |  |  |
| Pérez-Bárcena 2008 | 8(2) | 7(3) | NA | NA |  | guidelines of the American Society of | NA |  |  |  |  |  |  |
|  | NA |  | NA |  |  | Parenteral and Enteral Nutrition |  |  |  |  |  |  |  |
| Fuentes-Orozco 2008 | NA |  | NA | NA |  | NA | NA |  |  |  |  |  |  |
| Pérez-Bárcena 2010 | 7(3.1) | 7(3) | NA | NA |  | commenced between 24 and 48 hours after | NA |  |  |  |  |  |  |
|  |  |  |  |  |  | admittance to the ICU admitted to the ICU |  |  |  |  |  |  |  |
| Andrews 2011 | NA |  | NA | NA |  | started as soon as practicable with the | NA |  |  |  |  |  |  |

| **study** | **SOFA** | | **CRRT** | **Gln**  **(mmol/l)** | | **Time to add nutrition** | **Comorbidities** | | | | | | |
| --- | --- | --- | --- | --- | --- | --- | --- | --- | --- | --- | --- | --- | --- |
|  | **Gln** | **Con** |  |  |  |  | **renal** | **diarrhea** | **abdominal** | **gastric** | **Hypertrigly** | **Hypergly** | **liver** |
|  |  |  |  | **Gln** | **Con** |  | **failure** |  | **distention** | **residuals** | **ceridemia** | **cemia** | **disorder** |
|  |  |  |  |  |  | Pharmacy |  |  |  |  |  |  |  |
| Grau 2011 | 6(4-9) | 7(4-9) | 31/121 | NA |  | Patients received the first administration | NA |  |  |  |  |  |  |
|  |  |  |  |  |  | in the first 3 days of ICU admission |  |  |  |  |  |  |  |
| [Wernerman 2011](http://www.ncbi.nlm.nih.gov/pubmed?term=Wernerman%20J%5BAuthor%5D&cauthor=true&cauthor_uid=21658010) | 9 | 9 | NA | NA |  | infusion for 12 h daily during the ICU stay | NA |  |  |  |  |  |  |
| [Goeters 2002](http://www.ncbi.nlm.nih.gov/pubmed?term=Goeters%20C%5BAuthor%5D&cauthor=true&cauthor_uid=12352037) | NA |  | NA | 0.48 | 0.48 | within 3 days after admission to |  |  |  |  |  |  |  |
|  |  |  |  |  |  | ICU and before start of balanced nutritional | NA |  |  |  |  |  |  |
|  |  |  |  |  |  | therapy, indication for total parenteral |  |  |  |  |  |  |  |
|  |  |  |  |  |  | nutrition |  |  |  |  |  |  |  |
| [Wischmeyer 2001](http://www.ncbi.nlm.nih.gov/pubmed?term=Wischmeyer%20PE%5BAuthor%5D&cauthor=true&cauthor_uid=11700398) | NA |  | 18/26 | NA |  | on enteral feeding within 48 hrs of admission | NA |  |  |  |  |  |  |
| Heyland 2013 | 8.5(2.8) | 8.3(2.8) | 134/1217 | 0.495 | 0.481 | Canadian Critical Care Nutrition practice | NA |  |  |  |  |  |  |

**Additional file 5.Summary of the population included in the meta-analysis:**

NA: Not available
